# Increased Lung Catalase Activity Confers Protection Against Experimental RSV Infection

**DOI:** 10.1038/s41598-020-60443-2

**Published:** 2020-02-27

**Authors:** Maria Ansar, Teodora Ivanciuc, Roberto P. Garofalo, Antonella Casola

**Affiliations:** 1 University of Texas Medical Branch, Department of Microbiology and Immunology Galveston, Galveston, TX 77555 USA; 2 University of Texas Medical Branch, Department of Pediatrics, Galveston, TX 77555 USA; 3 University of Texas Medical Branch, Institute for Human Infections and Immunity, Galveston, TX 77555 USA

**Keywords:** Diseases, Infectious diseases, Viral infection

## Abstract

Respiratory syncytial virus (RSV) infection in mouse and human lung is associated with oxidative injury and pathogenic inflammation. RSV impairs antioxidant responses by increasing the degradation of transcription factor NRF2, which controls the expression of several antioxidant enzyme (AOE) genes, including catalase. Since catalase is a key enzyme for the dismutation of virus-mediated generation of hydrogen peroxide (H_2_O_2_) we developed a model of intranasal supplementation of polyethylene glycol-conjugated catalase (PG-CAT) for RSV-infected mice. The results of our study show that PG-CAT supplementation was able to increase specific enzymatic activity along with reduction in H_2_O_2_ in the airways and had a significant protective effect against RSV-induced clinical disease and airway pathology. PG-CAT treated mice showed amelioration in airway obstruction, reduction in neutrophil elastase and inflammation. Improved airway hyperresponsiveness was also observed in mice that received PG-CAT as a treatment post-viral inoculation. In addition, PG-CAT greatly reduced the concentration of inflammatory cytokines and chemokines, including IL-1, TNF-α, IL-9, CXCL1, CCL2, and CCL5 in the bronchoalveolar lavage fluid of RSV-infected mice, without increasing viral replication in the lung. In conclusion, catalase supplementation may represent a novel pharmacologic approach to be explored in human for prevention or treatment of respiratory infections caused by RSV.

## Introduction

Respiratory syncytial virus (RSV) bronchiolitis remains one of the most common respiratory infections in infants and young children. It resulted in an estimated 33.1 million cases of lower respiratory tract infection (LRTIs) globally in 2015, accounting for 3.2 million hospital admissions children^1^. There is significant morbidity and mortality associated with severe RSV bronchiolitis, particularly in developing countries^2^. While use of palivizumab, a monoclonal antibody, is fairly effective for RSV infection prophylaxis in high risk patients, current management is otherwise supportive^3^. Our understanding of the underlying molecular mechanisms responsible for RSV lower respiratory tract disease is still incomplete and remains a critical need to improve patient care and outcomes. Previous work in our laboratory has shown that RSV infection is associated with increased reactive oxygen species (ROS) production, leading to transcription factor activation and chemokine gene expression^4,5^. The transcription factor NF-E2-related factor 2 (NRF2), which binds the antioxidant response element (ARE) present in the promoter region of antioxidant enzymes (AOEs) plays a key role in the expression of AOEs^6^. NRF2 cellular levels decrease in the course of RSV infection *in vitro*^7^ and *in vivo*^8^, due to increased degradation^9,10^, leading to inhibition of AOE expression and cellular oxidative damage^7,8^, a process that has been implicated in the pathogenesis of acute and chronic airway diseases^11^.

Catalase, one of earliest known and best characterized of the AOEs is a homotetrameric protein (MWt., 240 kDa) that catalyzes the dismutation of peroxide to water and oxygen (equation: 2H_2_O_2_ → 2H_2_O + O_2_)^12^. Catalase is ubiquitous to most aerobic cells and in the lung is specially localized in alveolar type II pneumocytes and macrophages^13^. Catalase is the only antioxidant enzyme increased both at the mRNA and at the activity levels during human lung morphogenesis^14^. Indeed, catalase is considered to be the most important antioxidant enzyme consuming exogenous hydrogen peroxide in type II pneumocytes, which are the most resistant cell type in the lung^15,16^. We have previously shown that RSV inhibits expression of AOE in airway epithelial cells, which are the major site of viral replication^7^. In those studies A549 cells, a human alveolar type II-like epithelial cell line, and small airway epithelial (SAE) cells, normal human cells derived from terminal bronchioli, were infected with RSV and harvested at various time points to measure oxidation markers and AOE by quantitative real-time PCR, Western blot and bioactivity. While oxidative markers such as lipid peroxidation products were increased by infection, levels of AOE were significantly reduced compared to control uninfected cells, with catalase and SOD1 being dramatically reduced at peak of viral replication (48 h). Subsequent investigations of mouse BAL by two-dimensional gel electrophoresis, MALDI-TOF-MS spectrometry, Western blot and bioactivity, revealed that RSV infection induced a significant decrease in the expression and activity of AOEs, including catalase, SOD, glutathione S-transferase and glutathione peroxidase, which correlated with parameters of clinical disease and airway inflammation^8^. Of clinical relevance we also showed in a group of RSV-infected infants that levels of AOEs were reduced in nasopharyngeal secretions obtained from hypoxemic patients or in those requiring ventilatory support (i.e. more severe clinical disease)^8^. Since underlying genetic factors may also contribute to the overall pro-oxidative and anti-oxidative balance and consequent disease severity following RSV infection we conducted a study in a group of 115 infants and young children with RSV positive LRTI (62 categorized as mild, 36 moderate and 17 severe disease) and genotyped them for ten known alleles (SNPs) in six AOE genes and in the Nrf2 gene. From this analysis, we found that one SNP in the antioxidant enzyme catalase, *rs1001179*, which is mapped to the gene promoter region and has been shown to be functional with higher enzyme expression/activity, had a protective effect on disease severity: while present at the expected population level among patients with mild disease, it had lower frequency in those with moderately severe and was absent in patients with severe disease^17^.

Overall these findings suggest that RSV-induced cellular oxidative damage is the result of an imbalance between ROS production and antioxidant cellular defenses, particularly those associated with catalase-mediated H_2_O_2_ dismutation. Therefore, we questioned whether exogenous supplementation of catalase could ameliorate airway disease using an experimental mouse model of RSV infection. The results of our study presented herein demonstrate that catalase supplementation via the intranasal route, either prior to or soon after infection significantly ameliorated clinical parameters of RSV infection, including body weight loss and disease score, reduced mucosal inflammation and showed a remarkable effect on airway obstruction, a characteristic of early RSV infection in mice. We suggest that catalase supplementation represents a novel therapeutic opportunity to be explored in human studies for the prevention/treatment of RSV bronchiolitis. Some of the results of this study have previously been presented as an abstract^17^.

## Results

### Catalase treatment improves RSV disease in mice

**Figure 1:**
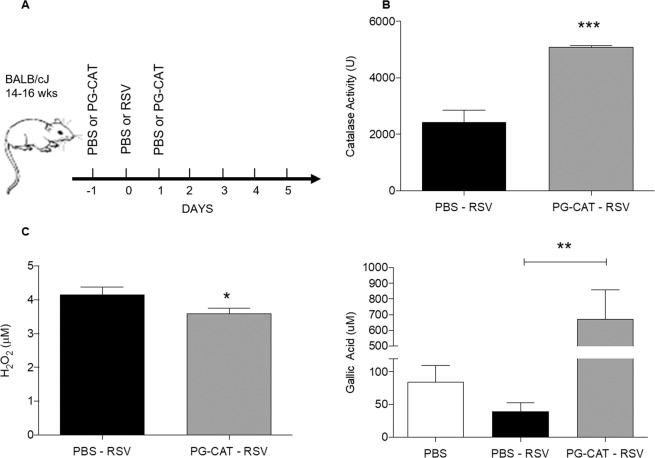
Catalase activity, hydrogen peroxide levels and antioxidant capacity in mice treated with PG-CAT. BALB/c mice were treated with PG-CAT or control (PBS), infected with RSV and BALF was collected at day 1 and 2 p.i. to measure: (**A**) Shows an outline of the treatment strategy utilized for these and all other experiments unless otherwise indicated. (**B**) Catalase activity, (n ≥ 7 per group) (**C**) H_2_O_2_ levels (n = 4), and (**D**) hydroxyl radical antioxidant capacity (n ≥ 3). Data are mean ± SEM. Statistics were determined using student’s *t-*test (**A–B**) and one-way ANOVA (**C**). Tukey’s multiple comparison analysis was used for inter-group analysis. ***p < 0.001, **p < 0.01, and *p < 0.05.

To determine whether increasing the levels of catalase in the airways would confer protection against RSV infection, we administered PG-CAT i.n. to mice infected with RSV (Fig. 1A). This catalase formulation was selected based on its enhanced bioavailability and prolonged activity in biological fluids thus resulting in lower dose requirements. We first determined whether PG-CAT treatment improved lung antioxidant capacity by administering the first dose (2.84 mg/kg) 24 h prior to RSV inoculation and a second dose (2.84 mg/kg) 24 post-infection (p.i.). Catalase activity was then determined in BALF at day 2 p.i. As expected, RSV-infected mice that were treated with PG-CAT had a significant higher level of catalase activity (~2 fold) compared to untreated mice (Fig. 1B). PG-CAT treatment had no effect on endogenous catalase mRNA expression per se (Supplementary Fig. 1). PG-CAT treatment resulted in a reduction in overall levels of H_2_O_2_ in BALF (Fig. 1C), and in a significant increase of airway antioxidant capacity, compared to infected untreated mice (RSV infection resulted in a ~2 fold reduction in antioxidant capacity compared to PBS) (Fig. 1D). The antioxidant capacity measured utilizing a gallic acid standard as reference represents the ability of the treatment to quench hydroxyl radicals, which are produced in RSV infection through interaction of hydrogen peroxide with neutrophil products i.e. myeloperoxidase^18^ An increase in this parameter indicates a significant increase in ROS quenching capacity.

**Figure 2:**
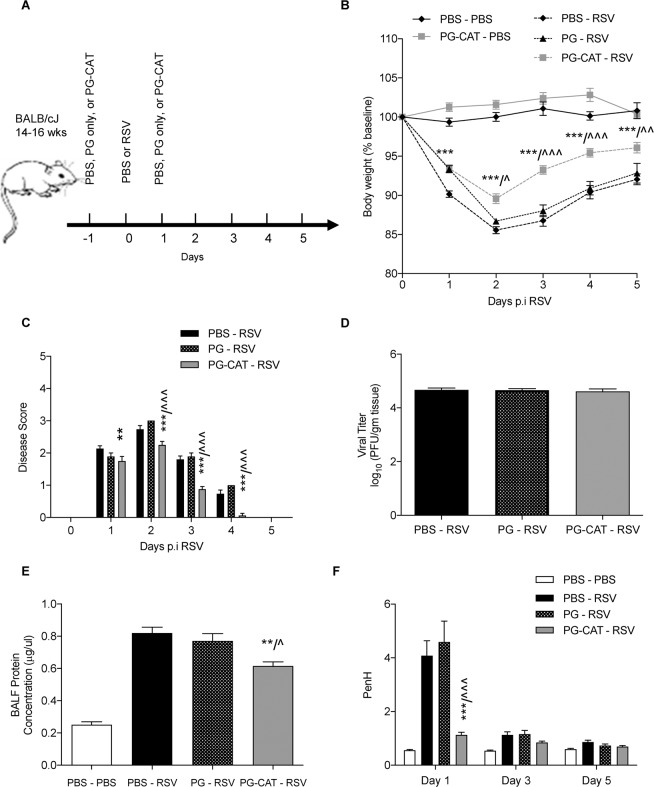
Effect of PG-CAT treatment on RSV disease in mice. BALB/c mice were either un-infected and treated with PBS or PG-CAT (PBS – PBS or PG-CAT – PBS), or infected and D0 and treated at D(−1) and D(1) with PBS, PG only, or PG-CAT (PBS – RSV, PG – RSV, and PG-CAT – RSV) (**A**). Mice were assessed for: body weight loss (**B**); clinical disease score (**C**); lung viral titers by plaque assays at day 5 p.i. (**D**); BALF total protein concentration at day 2 p.i. (E); airway obstruction by whole body plethysmography (**F**). Data are mean ± SEM. Significance was determined using repeated measures ANOVA (**B**, **C**, and **F**), one-way ANOVA (**E**) and student’s *t-*test (**D**). Tukey’s multiple comparison analysis was used for inter-group analysis. *p < 0.05, **p < 0.01, and ***p < 0.001 is significance for PBS – RSV vs PG-CAT – RSV. ^p < 0.05, ^^p < 0.01, and ^^^p < 0.001 is significance for PG – RSV v PG-CAT – RSV.

PG-CAT treatment (as in Fig. 2A) consistently resulted in a significant reduction in body weight loss (Fig. 2B) and improvement of clinical disease score (Fig. 2C), in particular after day 2 of RSV infection, with no significant effect on viral replication in the lung (Fig. 2D). In addition, PG-CAT treatment was associated with a significant reduction in RSV-induced BALF total protein levels, a measure of epithelial barrier damage (Fig. 2E). In agreement with the improved body weight loss, clinical disease and lung epithelial damage, PG-CAT treated mice showed a significant improvement in lung function, as shown by a reduction in airway obstruction (i.e., basal Penh values) measured by whole body plethysmography (Fig. 2F). Similar improvements in body weight loss and clinical disease were seen with a lower (1.42 mg/kg) and a higher (5.68 mg/kg) dose of PG-CAT (Supplementary Fig. 2). In addition, since PG alone could act as an anti-inflammatory molecule (14;15), we compared untreated vs PG-treated RSV-infected mice. There was no change with PG treatment in body weight loss, clinical disease score, protein concentration in BALF, or airway obstruction, apart from a small improvement in body weight loss at day 1 p.i. (Supplementary Fig. 3).

Additionally, disease parameters were also determined in a post-infection treatment model of PG-CAT administration. Although there was no significant change observed in body weight loss (supplementary Fig. 4A), or clinical disease (Supplementary Fig. 4B), PG-CAT treatment resulted in a significant improvement in airway obstruction (Supplementary Fig. 4C), and AHR (Supplementary Fig. 4D).

### Effect of catalase treatment on lung inflammation and histopathology

**Figure 3:**
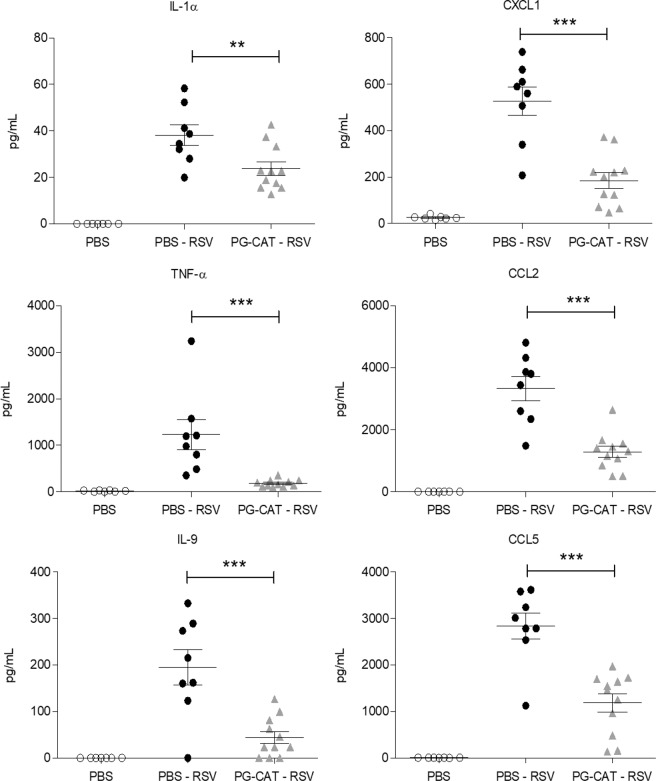
Inflammatory cytokines and chemokines in BALF. BALB/c mice were treated with PG-CAT and infected with RSV. BALF was obtained at day 2 p.i. to determine levels of cytokines and chemokines by multiplex assay. Data are mean ± SEM. Significance was determined using one-way ANOVA (**A–F**). Tukey’s multiple comparison analysis was used for inter-group analysis. ***p < 0.001, **p < 0.01, and *p < 0.05.

**Figure 4:**
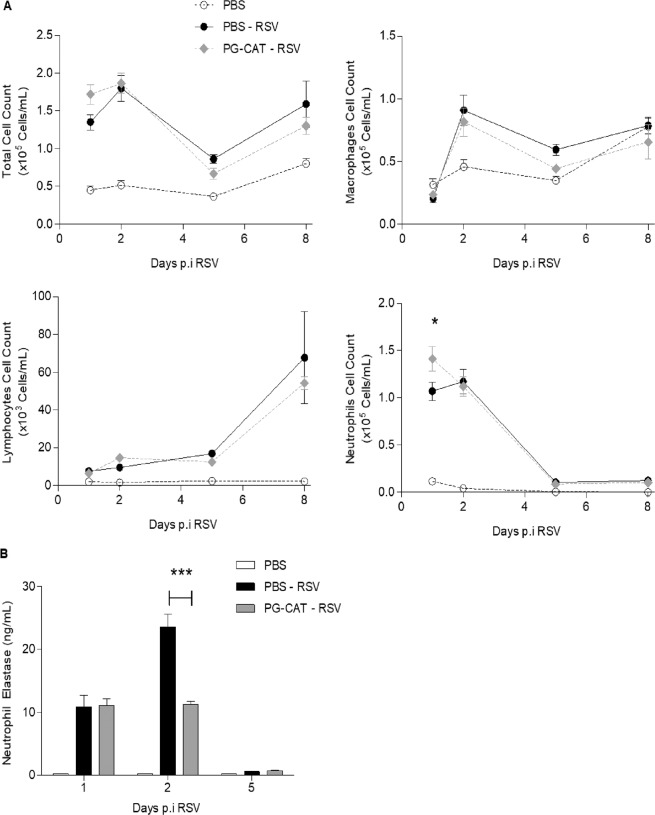
Effect of PG-CAT treatment on BALF cell recruitment. BALB/c mice were treated with PG-CAT and infected with RSV. Total and differential cell count in BALF was done at day 1, 2, 5 and 8 p.i. (**A**). Neutrophil elastase levels were measured in BALF at day 1, 2 and 5 p.i. (**B**). Data are mean ± SEM. Significance was determined using repeated measures ANOVA (**A**) and one-way ANOVA (**B**). Tukey’s multiple comparison analysis was used for inter-group analysis. *p < 0.05, and ***p < 0.001.

**Figure 5:**
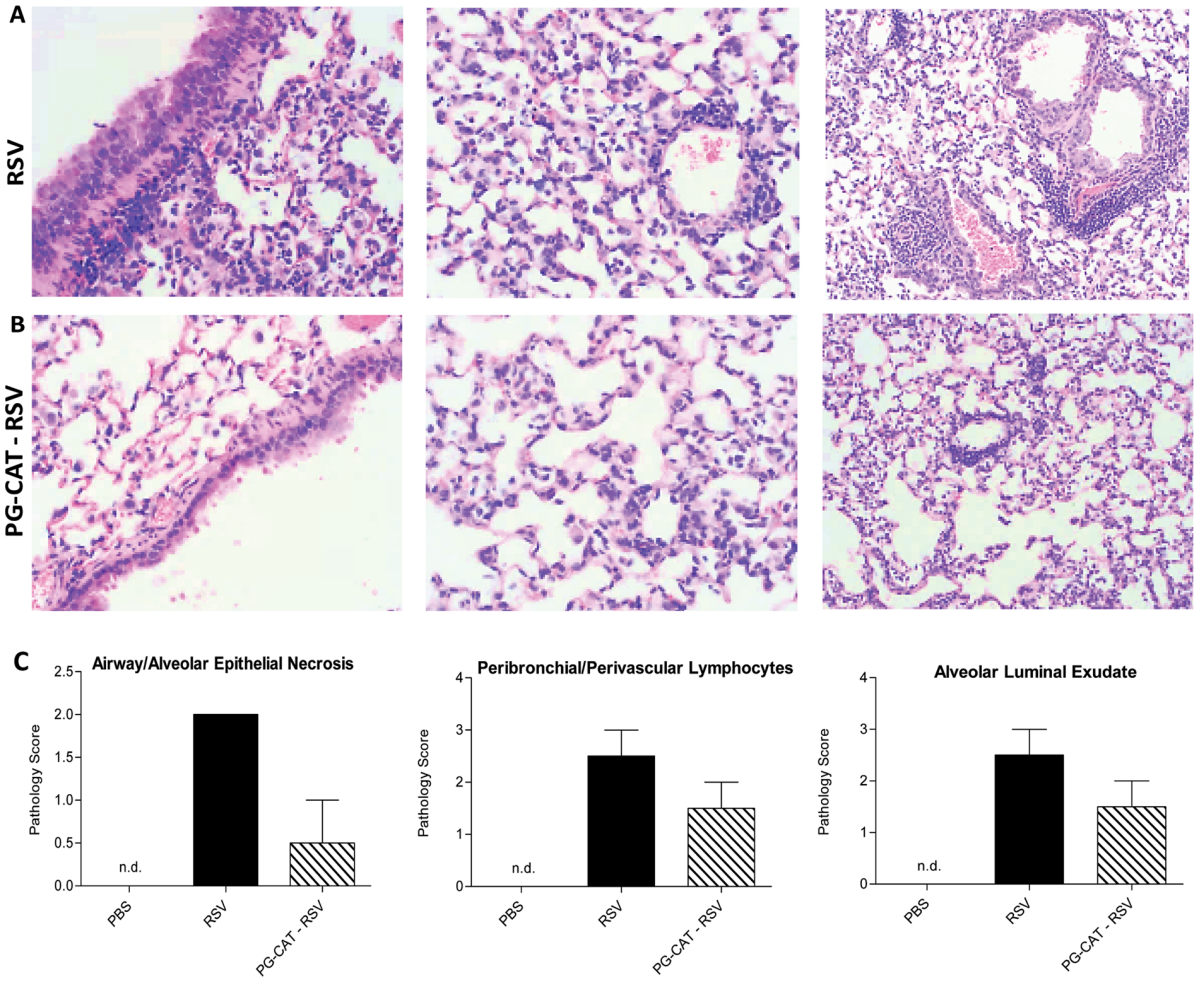
PG-CAT treatment reduces lung pathology. BALB/c mice were treated with PG-CAT and infected with RSV. Lungs were harvested at day 3 p.i and imbedded in formalin for sectioning. Slides were prepared with H&E staining and images are shown as RSV (**A**), PG-CAT-RSV (**B**), and pathology score (**C**, mean ± SEM).

As inflammation is an important component of RSV-induced lung disease, we investigated BALF cytokine and chemokine levels and inflammatory cell recruitment in response to PG-CAT administration (as in Fig. 2A). There was a significant reduction in RSV-induced secretion of the cytokines IL-1α, TNF-α and IL-9, as well as of the chemokines CXCL1, CCL2 and CCL5 at day 2 p.i. in mice treated with PG-CAT, compared to untreated/infected mice (Fig. 3). Interestingly, most of the RSV-induced cytokines and chemokines measured in BALF at day 1 p.i. were not significantly affected by PG-CAT (see all data in supplementary Tables 1 and 2). In regard to inflammatory cell recruitment, there was no significant change in total cell count in BALFs of infected mice treated with PG-CAT, compared to infected untreated mice, at all the time points tested. Numbers of macrophages, neutrophils and lymphocytes were also similar between the two groups, with the exception of a small increase in neutrophil count at day 1 p.i. in mice treated with PG-CAT compared to untreated (Fig. 4A). Since we noticed that neutrophils in the PG-CAT group exhibited some features of immaturity (banded nuclei, retention of granules, etc.), we determined whether PG-CAT treatment altered neutrophil activation by assessing neutrophil elastase levels in BALF. There was no significant difference in elastase at day 1 p.i., however levels were significantly decreased on day 2 p.i. in PG-CAT treated mice, compared to untreated mice (Fig. 4B), consistently with the reduction by PG-CAT of neutrophil activating chemokines observed at day 2 (Fig. 3). Histopathology analysis of H&E stained lung sections showed that PG-CAT treatment resulted in reduced airway epithelial necrosis, interstitial pneumonia, and reduced damage to airway architecture (Fig. 5). Uninfected mice had no lung microscopic lesions.

## Discussion

Catalase is an antioxidant enzyme present in most aerobic cells that catalyzes dismutation of hydrogen peroxide to water and oxygen^12^. In the lung, catalase activity increases in parallel with the its embryologic development and at full maturity it is specially localized in alveolar type II pneumocytes and alveolar macrophages, both cell types being a primary target of respiratory viral entry and replication^13^. While certain stimuli such as hyperoxia and cytokines induce catalase expression/activity, other stimuli like prolonged cigarette smoke exposure can lead to decreased catalase mRNA and protein levels^19,20^. In addition, genetic factors such as polymorphisms in the catalase gene promoter have been associated with increased basal expression of catalase gene, which may also contribute to an enhancement in the overall anti-oxidative capacity^21^.

Our data in RSV-infected mice demonstrate that two single doses of intranasal PG-CAT effectively increased levels of lung catalase and its antioxidant capacity, and reduced disease, as body weight loss, clinical illness score, pathology, and airway obstruction were all significantly improved compared to untreated control littermates. In addition, PG-CAT supplementation at a time subsequent to RSV inoculation resulted also in an improvement of RSV-induced airway obstruction measured at day 1 post-infection and AHR measured at a later time point of infection (day 5), but not clinical disease or body weight loss. A possible explanation is that unlike natural infection in human which starts in the upper airways, the experimental RSV infection of mice is characterized by a rapid delivery of the virus inoculum to the lower airways. Thus, all the post-infection treatment modalities are less or no effective at all in the modulation of early inflammatory cytokine production that control lung inflammation and clinical disease. Indeed, while in the post-infection catalase treatment we did not find any reduction of key inflammatory cytokines that may be associated with viral-induced clinical disease in the mouse model, it was nonetheless effective in improving airway reactivity that we believe is affected by oxidative pathways associated with RSV-induced production of H_2_O_2_. The possibility of using catalase as a treatment in RSV infection in human is however speculative at this point as the biologic significance of such airway reactivity improvement in mice remains to be determined for human airway physiology. In other animal models of pulmonary diseases, including asbestos- and radiation-dependent pulmonary fibrosis, hyperoxia, hydrogen peroxide-mediated injury, and viral infection with influenza H1N1, catalase treatment has also been shown to be effective in modulating clinical illness^22–25^. Although the exact mechanism(s) underlying the disease-improving effect of PG-CAT treatment remains to be established, our data suggest a few possibilities. First, PG-CAT administration was associated with significant reduction in overall levels of H_2_O_2_ and in a significant increase of airway antioxidant capacity in BALF of mice infected with RSV. ROS generated in the course of inflammatory processes are known to play a pathogenic role in lung diseases such as asthma and COPD^15^, and increasing lung antioxidant level protects against severe disease in a mouse model of RSV infection^26^. H_2_O_2_ per se is known to regulate airway smooth muscle tone, leading to increased contraction and enhanced airway resistance^27–30^. In addition, H_2_O_2_ also enhances epithelial barrier leakage via an increase in vascular permeability and damage to epithelial actin cytoskeleton^28,31^. Therefore, improvements observed in PG-CAT treated animals could be linked to an improved capacity to scavenge H_2_O_2._

In addition, PG-CAT treatment resulted in significant inhibition of several RSV-induced inflammatory cytokines and chemokines, as early as day 2 post-infection. TNF-α, which was severely reduced in PG-CAT treated mice, has been correlated with enhance body weight loss, clinical disease, pathology and airway hyperreactivity in RSV-infected mice and children^32,33^. Chemokines produced during the course of RSV infection have been linked to disease severity, including CXCL1, CCL2 and CCL5, either in murine models of infection or in patients. High CXCL1 levels was correlated with hypoxia and increased ventilation needs^34,35^. Similarly CCL2, CCL3 and CCL4 were linked to enhanced disease severity, being higher in patients requiring admission to intensive care in patients^36^. Genetic variants of the CCL5 major receptor was also linked to RSV diseases severity in patients and to enhancement of allergic airway reactivity in murine models of infection^37^.

Neutrophils recruitment to the airways has also been associated with enhanced pathology in the course of RSV infection^38–40^. Neutrophil products, i.e., elastase and CXCL1, are strongly linked to enhanced disease severity in numerous models of lung inflammation including acute edematous injury, bleomycin-induced acute lung injury, smoke inhalation and pneumonia^41–44^. High levels of TNF-α and CXCL1 are associated with enhanced elastase release from neutrophils^45^, both of which were reduced by PG-CAT treatment. Altogether, catalase administration altered several components of the inflammatory response to RSV infection, which could account for the observed clinical improvement.

In summary, our results suggest that the antioxidant catalase exerts a protective role in the context of RSV infection. Increasing airway catalase levels at the time of RSV infection could represent a potential novel therapeutic approach to ameliorate viral-induced lung disease.

## Methods

### Ethics statement

Animal care and use were carried out in accordance with the recommendations in the Guide for the Care and Use of Laboratory Animals of the National Institutes of Health. The protocol was approved by the Institutional Animal Care and Use Committee of the University of Texas Medical Branch at Galveston (Protocol: 9001002). The mice were euthanized by an intraperitoneal injection of ketamine and xylazine and exsanguinated via the femoral vessels.

### Mice and RSV infection protocol

RSV Long strain was grown in HEp-2 cells and purified by centrifugation on discontinuous sucrose gradients, as described^46^, and viral pools were titered in plaque forming units (PFU)/mL using a methylcellulose plaque assay, as described^47^. Female BALB/c mice (14–16 weeks old) were purchased from Jackson Laboratory (Bar Harbor, ME). Mice were lightly anesthetized with 0.1 mL of Ketamine/xylazine (200/70 mg/mL) injected intraperitoneally. Mice were then treated intranasally (i.n.) with control phosphate buffered saline (PBS), or with polyethylene glycol-catalase [(PG-CAT, Sigma, St. Louis, MO, 62.5 μg (2.84 mg/kg)] or with polyethylene glycol (PG) only (2.19 mg/kg). For 0.5x and 2x doses mice were treated with 31.25 μg (1.42 mg/kg) and 125 μg (5.68 mg/kg) of PG-CAT, respectively. Twenty-four hours later, mice were infected with 5 × 10^6^ PFU of RSV diluted in 50 μl of PBS (groups are identified as PBS-RSV, PG-RSV and PG-CAT-RSV; the uninfected/untreated control group is indicated as PBS and the uninfected/PG-CAT control is indicated as PG-CAT-PBS). The following day, mice were given one additional dose of PBS, PG or PG-CAT. For the treatment model mice were infected with RSV and treated with either PBS or 62.5 μg of PG-CAT at 3 h and D3 p.i.

### Clinical disease

Animals from all groups were evaluated on a daily basis for weight loss, illness score, and presence of any respiratory symptoms. The percentage of body weight change was plotted over time. A clinical illness score for mice was used to measure the severity of clinical disease (0-healthy; 1-barely ruffled fur; 2-ruffled fur but active; 3-ruffled fur and inactive; 4-ruffled fur, inactive, and hunched; and 5-dead). These parameters have been shown to closely correlate with lung pathology in experimental infection of mice^26,48^. For clinical disease parameters groups consisted of PBS - PBS (n = 9), PG-CAT - PBS (n = 11), PBS-RSV (n = 15), PG-RSV (n = 9), and PG-CAT-RSV (n = 16). For dose-response studies of clinical disease, data were collected from groups of mice treated with 0.5x PG-CAT - RSV (n = 8) and 2x PG-CAT - RSV (n = 8). RSV viral titers were determined using a plaque assay with increasing dilution of lung lysate (1:2–1:256), grown on a monolayer of HEp2 cells for five days (n = 8).

### Broncholaveolar lavage (BAL)

BAL fluid (BALF) was collected *via* the trachea by flushing the lungs twice with 1 ml of ice-cold PBS. A total of 100 µl of BAL fluid was used for cytospin analysis, and the rest was immediately centrifuged and stored at −80 °C for cytokine analysis. Total number of BAL cells was counted with a hemacytometer and viability was assessed by trypan blue. BAL differential cell counts were determined using morphogenic criteria under light microscopy of Protocol Hema-3 (Fisher Scientific) stained cytospins with a total count of 300 cells per slide^26,47^. Data were collected at D1, D2, D5, and D8 (n ≥ 8 at D1 and D2 and n ≥ 4 at D8). Neutrophil elastase was determined using BAL samples at D1 (n = 4), D2 (n ≥ 8), and D5 (n = 4), using a neutrophil elastase ELISA (R&D Systems). Protein concentration of BAL was determined utilizing a bovine serum albumin standard (Sigma). Groups consisted of PBS-control (n = 7), RSV (n = 8), PG-RSV (n = 8), and PG-CAT – RSV (n = 8).

### Measurement of cytokines, and chemokines

Levels of cytokines and chemokines in BAL fluid were determined with the Bio-Plex Pro Mouse Group I, 23-plex panel (Bio-Rad Laboratories, Hercules, CA, USA), according to the manufacturer’s instructions^49^. Groups included PBS (n = 2), RSV (n = 11), PG-RSV (n = 10), and PG-CAT – RSV (n = 12) for day 1, and PBS (n = 7), RSV (n = 8), PG-RSV (n = 11), and PG-CAT – RSV (n = 11) for day 2. The panel included the following cytokines with the lower limit of quantitation (LLQ): IL-1α (1.84 pg/ml), IL-1β (10.36 pg/ml), IL-2 (3.72 pg/ml), IL-3 (1.55 pg/ml), IL-4 (6.98 pg/ml), IL-5 (3.57 pg/ml), IL-6 (0.74 pg/ml), IL-9 (6.89 pg/ml), IL-10 (2.95 pg/ml), IL-12 p40 (1.53 pg/ml), IL-12 p70 (1.62 pg/ml), IL-13 (47.2 pg/ml), IL-17 (2.65 pg/ml), granulocyte-macrophage colony-stimulating factor (GM-CSF) (21.2 pg/ml), gamma interferon (IFN-γ) (1.84 pg/ml), tumor necrosis factor alpha (TNF-α) (5.8 pg/ml), G-CSF (5.1 pg/ml), Eotaxin (257.9 pg/ml), KC (3.2 pg/ml), MCP-1 (22.4 pg/ml), macrophage inflammatory protein 1α (MIP-1α) (256.2 pg/ml), MIP-1β (3.33 pg/ml) and RANTES (2.78 pg/ml). Data is presented as a mean of three experiments (PBS n = 7, and infected groups n ≥ 8).

### Airway obstruction and hyperresponsiveness (AHR)

Airway obstruction and AHR were assessed in unrestrained mice at different times after infection using whole-body barometric plethysmography (Buxco, DSI, New Brighton, MN) to record enhanced pause (Penh)^50,51^. Penh is a dimensionless value that represents a function of the ratio of peak expiratory flow to peak inspiratory flow and a function of the timing of expiration. Airway obstruction is measured as baseline Penh (no challenge with methacholine). Airway obstruction was measured at D1, 3, and 5 (n = 7) for 5 min to obtain Penh values. AHR was determined at D5 following challenge when increasing doses of methacholine of nebulized methacholine (3.25, 6.25, 12.5, 25, and 50 mg/ml) for 2 min, and data were recorded for another 3 min.

### Pulmonary histopathology

Mice were euthanized and the entire lung was perfused, removed, and fixed in 10% buffered formalin following by paraffin embedding. Multiple 4-µm longitudinal cross-sections were stained with hematoxylin and eosin (H&E). The slides were analyzed under light microscopy by a board-certified pathologist with expertise in mouse lung, unaware of the infection/treatment status of the animals. The pathologist assessed groups based on microscopic lesions in the lung, percentage of abnormal lung field, necrosis of epithelium and alveoli, presence of exudate (neutrophils, macrophages, and lymphocytes), and pneumocyte hypertrophy. Based on these parameters, slides were scored on a 0–3 scale (0 = normal, 1 = mild, 2 = moderate, and 3 = severe disease).

### Catalase, hydrogen peroxide and hydroxyl radical antioxidant capacity measurements

A catalase activity assay was utilized to measure catalase activity in mouse BALF, after protein/volume normalization (n ≥ 7). Catalase mRNA was determined through qRT-PCR (n = 3). Hydrogen peroxide (H_2_O_2_) levels (n = 4) and hydroxyl radical antioxidant capacity (HORAC) (n = 4) were determined in BALF samples using the hydrogen peroxide/peroxidase assay kit and the (HORAC) activity assay kit and analyzed with the FLUOstar Optima (BMG Labtech, Cary, NC). All kits were obtained from Cell Biolabs, San Diego, CA.

### Statistical analysis

For the animal studies, data were evaluated using repeated measures ANOVA, one-way ANOVA, and two-tailed unpaired Student’s *t*-test using GraphPad Prism 5.02 (GraphPad Software, Inc., San Diego, CA). A p value < 0.05 was considered significant and significance is designated with the symbol (*) representing ***p < 0.001, **p < 0.01 and *p < 0.05. All data is shown as a mean ± SEM.

## Supplementary information

Supplementary Legends.

Supplementary Table 1.

Supplementary Table 2.

Supplementary Figure 1.

Supplementary Figure 2.

Supplementary Figure 3.

Supplementary Figure 4.

## Data Availability

The datasets generated during and/or analyzed during the current study, and not submitted as supplementary material, are available from the corresponding author on reasonable request.

## References

[1] Shi, T. *et al*. Global, regional, and national disease burden estimates of acute lower respiratory infections due to respiratory syncytial virus in young children in 2015: a systematic review and modelling study, **390**(10098), 946 (2017). 10.1016/S0140-6736(17)30938-8 PMC5592248 28689664

[2] Nair, H. *et al*. Global burden of acute lower respiratory infections due to respiratory syncytial virus in young children: a systematic review and meta-analysis, **375**(9725), 1545 (2010). 10.1016/S0140-6736(10)60206-1 PMC2864404 20399493

[3] Palivizumab, a humanized respiratory syncytial virus monoclonal antibody, reduces hospitalization from respiratory syncytial virus infection in high-risk infants. The IMpact-RSV Study Group. *Pediatrics* **102**(3 Pt 1), 531 (1998). 9724660

[4] Casola A (2001). Oxidant tone regulates RANTES gene transcription in airway epithelial cells infected with Respiratory Syncytial Virus: role in viral-induced Interferon Regulatory Factor activation. J. Biol. Chem..

[5] Liu T (2004). Reactive oxygen species mediate virus-induced STAT activation: role of tyrosine phosphatases. J. Biol. Chem..

[6] Li W, Kong AN (2009). Molecular mechanisms of Nrf2-mediated antioxidant response. Mol. Carcinog..

[7] Hosakote YM (2009). Respiratory syncytial virus induces oxidative stress by modulating antioxidant enzymes. Am. J. Respir. Cell. Mol. Biol..

[8] Hosakote, Y. M. *et al*. Viral-mediated inhibition of antioxidant enzymes contributes to the pathogenesis of severe respiratory syncytial virus bronchiolitis, **183**(1), 1550 (2011). 10.1164/rccm.201010-1755OC PMC3137144 21471094

[9] Komaravelli, N. *et al*. Respiratory syncytial virus induces NRF2 degradation through a promyelocytic leukemia protein- ring finger protein 4 dependent pathway, **113**, 494 (2017). 10.1016/j.freeradbiomed.2017.10.380 PMC5699968 29107745

[10] Komaravelli N (2015). Respiratory syncytial virus infection down-regulates antioxidant enzyme expression by triggering deacetylation-proteasomal degradation of NRF2. Free Radic. Biol. Med..

[11] Ciencewicki J, Trivedi S, Kleeberger SR (2008). Oxidants and the pathogenesis of lung diseases. J. Allergy Clin. Immunol..

[12] Fridovich I (1986). Biological effects of the superoxide radical. Arch. Biochem. Biophys..

[13] Kinnula VL, Crapo JD (2003). Superoxide dismutases in the lung and human lung diseases. Am. J. Respir. Crit. Care. Med..

[14] Asikainen TM (1998). Expression and developmental profile of antioxidant enzymes in human lung and liver. Am. J. Respir. Cell Mol. Biol..

[15] Rahman I, Biswas SK, Kode A (2006). Oxidant and antioxidant balance in the airways and airway diseases. Eur. J. Pharmacol..

[16] Simon RH, DeHart PD, Nadeau DM (1989). Resistance of rat pulmonary alveolar epithelial cells to neutrophil- and oxidant-induced injury. Am. J. Respir. Cell. Mol. Biol..

[17] Jeffrey, C. *et al*. A polymorphism in the catalase gene promoter confers protection against severe RSV bronchiolitis, **141**(2, Supplement), AB10 (2018). 10.3390/v12010057 PMC7019864 31947722

[18] Garofalo RP, Kolli D, Casola A (2013). Respiratory syncytial virus infection: mechanisms of redox control and novel therapeutic opportunities. Antioxid. Redox. Signal..

[19] Betsuyaku T (2013). Bronchiolar epithelial catalase is diminished in smokers with mild COPD. Eur. Respir. J..

[20] Rahman I (2005). Redox signaling in the lungs. Antioxid. Redox. Signal..

[21] Kodydkova J (2014). Human catalase, its polymorphisms, regulation and changes of its activity in different diseases. Folia Biol. (Praha).

[22] Holm, B. A. *et al*., Mechanisms of H_2_O_2_-mediated injury to type II cell surfactant metabolism and protection with PEG-catalase, **261**, C751–C757 (1991). 10.1152/ajpcell.1991.261.5.C751 1951666

[23] Jacobson, J. M. *et al*. Antioxidants and antioxidant enzyme protection against pulmonary oxygen toxicity in the rabbit, *J. Appl Physiol.*, 1252 (1990). 10.1152/jappl.1990.68.3.1252 2341349

[24] Machtay, M. *et al*. Systemic polyethyelene glycol-modified (PEGylated) superoxide dismuatase and catalase mixture attenuates raidation pulmonary fibrosis in the C57/bl6 mouse, **81**, 196 (2006). 10.1016/j.radonc.2006.09.01 PMC1764603 17069914

[25] Murthy S (2009). Modulation of reactive oxygen species by Rac1 or catalase prevents asbestos-induced pulmonary fibrosis. Am J Physiol Lung Cell. Mol. Physiol.

[26] Castro SM (2006). Antioxidant treatment ameliorates respiratory syncytial virus-induced disease and lung inflammation. Am. J. Respir. Crit. Care Med..

[27] Kojima K (2007). Direct effects of hydrogen peroxide on airway smooth muscle tone: roles of Ca2+ influx and Rho-kinase. Eur. J. Pharmacol..

[28] Lee KS (2006). Hydrogen peroxide induces vascular permeability via regulation of vascular endothelial growth factor. Am J Respir Cell Mol Biol.

[29] Matyas, S., Pucovsky, V. & Bauer, V. Effects of various reactive oxygen species on the guinea pig trachea and its epithelium, **88**(3), 270 (2002). 10.1254/jjp.88.270 11949881

[30] Rhoden, K. J. & Barnes, P. J. Effect of hydrogen peroxide on guinea-pig tracheal smooth muscle *in vitro*: role of cyclooxygenase and airway epithelium, **98**(1), 325 (1989). 10.1111/j.1476-5381.1989.tb16898.x PMC1854660 2508982

[31] Boardman KC (2003). Actin re-distribution in response to hydrogen peroxide in airway epithelial cells. J. Cell. Physiol..

[32] Hussell T, Pennycook A, Openshaw PJ (2001). Inhibition of tumor necrosis factor reduces the severity of virus- specific lung immunopathology. Eur. J. Immunol..

[33] Puthothu B (2009). Association of TNF-alpha with severe respiratory syncytial virus infection and bronchial asthma. Pediatr Allergy Immunol.

[34] Bermejo-Martin JF (2007). Predominance of Th2 cytokines, CXC chemokines and innate immunity mediators at the mucosal level during severe respiratory syncytial virus infection in children. Eur. Cytokine Netw..

[35] Bont L (1999). Peripheral blood cytokine responses and disease severity in respiratory syncytial virus bronchiolitis. Eur Respir J.

[36] Tabarani, C. M. *et al*. Novel inflammatory markers, clinical risk factors and virus type associated with severe respiratory syncytial virus infection, **32**(12), 437 (2013). 10.1097/INF.0b013e3182a14407 PMC3883981 23804121

[37] Hull J (2003). Variants of the chemokine receptor CCR5 are associated with severe bronchiolitis caused by respiratory syncytial virus. J Infect. Dis..

[38] Geerdink RJ (2015). Neutrophils in respiratory syncytial virus infection: a target for asthma prevention. J. Allergy Clin. Immunol..

[39] Jaovisidha P (1999). Respiratory syncytial virus stimulates neutrophil degranulation and chemokine release. J. Immunol..

[40] Wang SZ (1998). Neutrophils induce damage to respiratory epithelial cells infected with respiratory syncytial virus,. Eur Respir J.

[41] Dunn JLM (2018). Blocking CXCL-1 dependent neutrophil recruitment prevents immune damage and reduces pulmonary bacterial infection after inhalational injury. Am. J. Physiol..

[42] Greene C (2003). Local impairment of anti-neutrophil elastase capacity in community-acquired pneumonia. J. Infect. Dis..

[43] Honore S (2004). Beneficial effect of an inhibitor of leukocyte elastase (EPI-hne-4) in presence of repeated lung injuries. Shock.

[44] Polverino E (2017). The role of neutorphil elastase inhibitors in lung diseases. Chest.

[45] Taggart C (2000). Increased elastase release by CF neutrophils is mediated by tumor necrosis factor-alpha and interleukin-8. Am J Physiol Lung Cell Mol Physiol.

[46] Ueba O (1978). Respiratory syncytial virus: I. concentration and purification of the infectious virus. Acta. Med. Okayama.

[47] Kisch AL, Johnson KM (1963). A plaque assay for respiratory syncytial virus. Proc. Soc. Exp. Biol. Med..

[48] Ivanciuc T (2016). Hydrogen sulfide is an antiviral and antiinflammatory endogenous gasotransmitter in the airways. Role in respiratory syncytial virus infection. Am. J. Respir. Cell Mol. Biol..

[49] Guerrero-Plata A, Casola A, Garofalo RP (2005). Human metapneumovirus induces a profile of lung cytokines distinct from that of respiratory syncytial virus. J Virol.

[50] Hamelmann E (1997). Noninvasive measurement of airway responsiveness in allergic mice using barometric plethysmography. Am. J. Respir. Crit Care Med..

[51] Lee JJ (1997). Interleukin-5 expression in the lung epithelium of transgenic mice leads to pulmonary changes pathognomonic of asthma. J. Exp. Med..

