# Supplementary material for: Increased Lung Catalase Activity Confers Protection Against Experimental RSV Infection
Source: Sci Rep. 2020 Feb 27;10:3653. doi: 10.1038/s41598-020-60443-2 (PMC7046725; doi:10.1038/s41598-020-60443-2)
Supplement: Supplementary file 1 — Supplementary Legends. [file 41598_2020_60443_MOESM1_ESM.docx]

**Supplementary Figure 1**. *Effect of PG-CAT treatment on endogenous catalase expression.* BALB/c mice were treated with PG-CAT and infected with RSV (treatment at D(-1) and D1 and infection at D0). Lungs were harvested for RNA extraction and catalase mRNA expression was determined at day 1 p.i by real-time PCR. Data are mean ± SEM. Statistical analysis was performed using one-way ANOVA.

**Supplementary Figure 2**. *PG-CAT dose response and RSV disease parameters.* BALB/c mice were treated with either control (PBS) or PG-CAT (0.5x - 1.42mg/kg, 1x - 2.84 mg/kg, and 2x - 5.68 mg/kg) (at D(-1) and D1), infected with RSV (at D0) and assessed for (A) body weight loss and (B) clinical disease score. Data are mean ± SEM. Significance determined using repeated measures ANOVA (A-B). Tukey’s multiple comparison analysis was used for inter-group analysis. * p<0.05, ** p<0.01, and *** p<0.001

**Supplementary Figure 3**. *Effect of PG alone on RSV disease parameters.* BALB/c mice were treated with either control (PBS) or PG alone, infected with RSV and assessed for (A) body weight loss, (B) clinical disease score, (C) total protein in BALF at day 2 p.i, and (D) airway obstruction by total body plethysmography. Data are mean ± SEM. Significance determined using repeated measures ANOVA (A-B, D) and one-way ANOVA (C). *** p<0.001.

**Supplementary Figure 4.** *Effect of PG-CAT post-infection treatment on RSV disease in mice.* BALB/c mice were infected with RSV and treated with PG-CAT at 3h and D3 p.i as shown in the graphic. Mice were assessed for: (A) body weight loss; (B) clinical disease score (C); baseline airway obstruction; and (D) airway hyperresponsiveness via whole body plethysmography. Data are mean ± SEM. Significance was determined using repeated measures ANOVA. Tukey’s multiple comparison analysis was used for inter-group analysis. * p<0.05, and *** p<0.001.
