# Supplementary figures and images for: Increased Lung Catalase Activity Confers Protection Against Experimental RSV Infection
Source: Sci Rep. 2020 Feb 27;10:3653. doi: 10.1038/s41598-020-60443-2 (PMC7046725; doi:10.1038/s41598-020-60443-2)

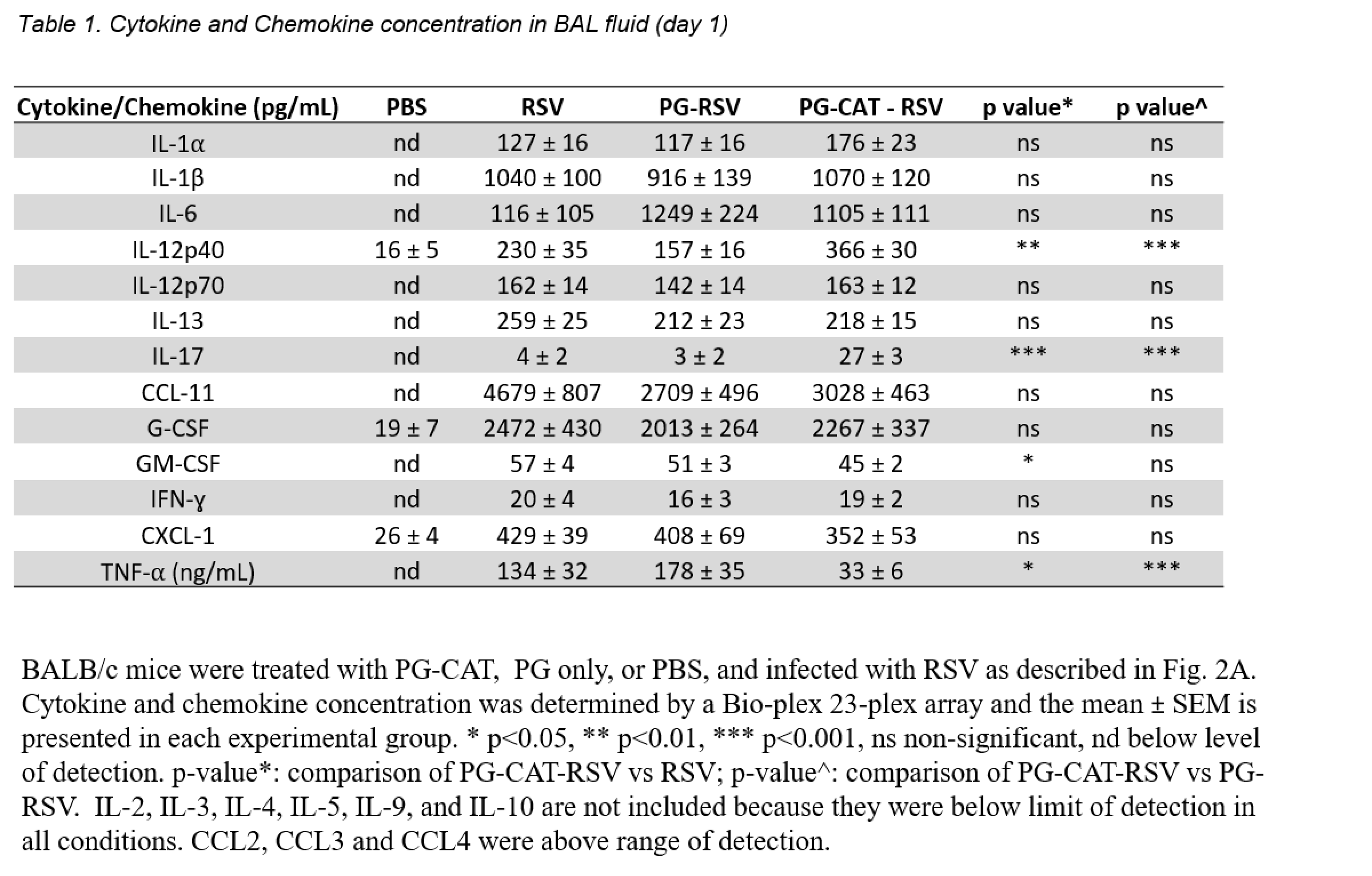

Supplement: Supplementary file 2 — Supplementary Table 1. [file 41598_2020_60443_MOESM2_ESM.tif]

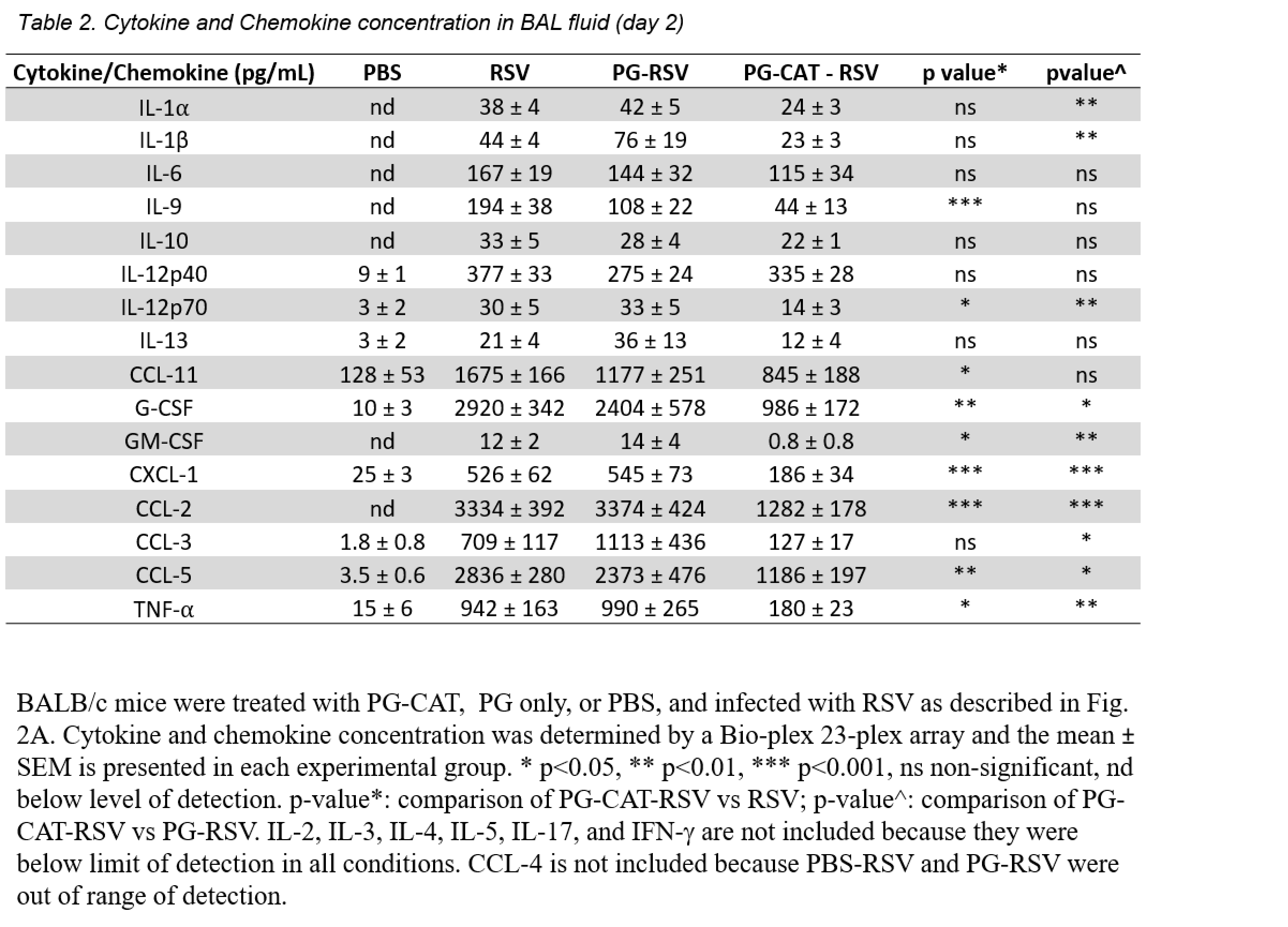

Supplement: Supplementary file 3 — Supplementary Table 2. [file 41598_2020_60443_MOESM3_ESM.tif]

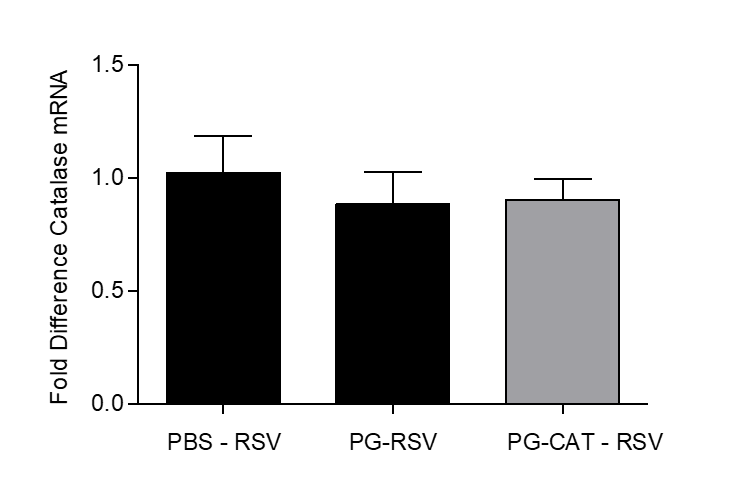

Supplement: Supplementary file 4 — Supplementary Figure 1. [file 41598_2020_60443_MOESM4_ESM.tif]

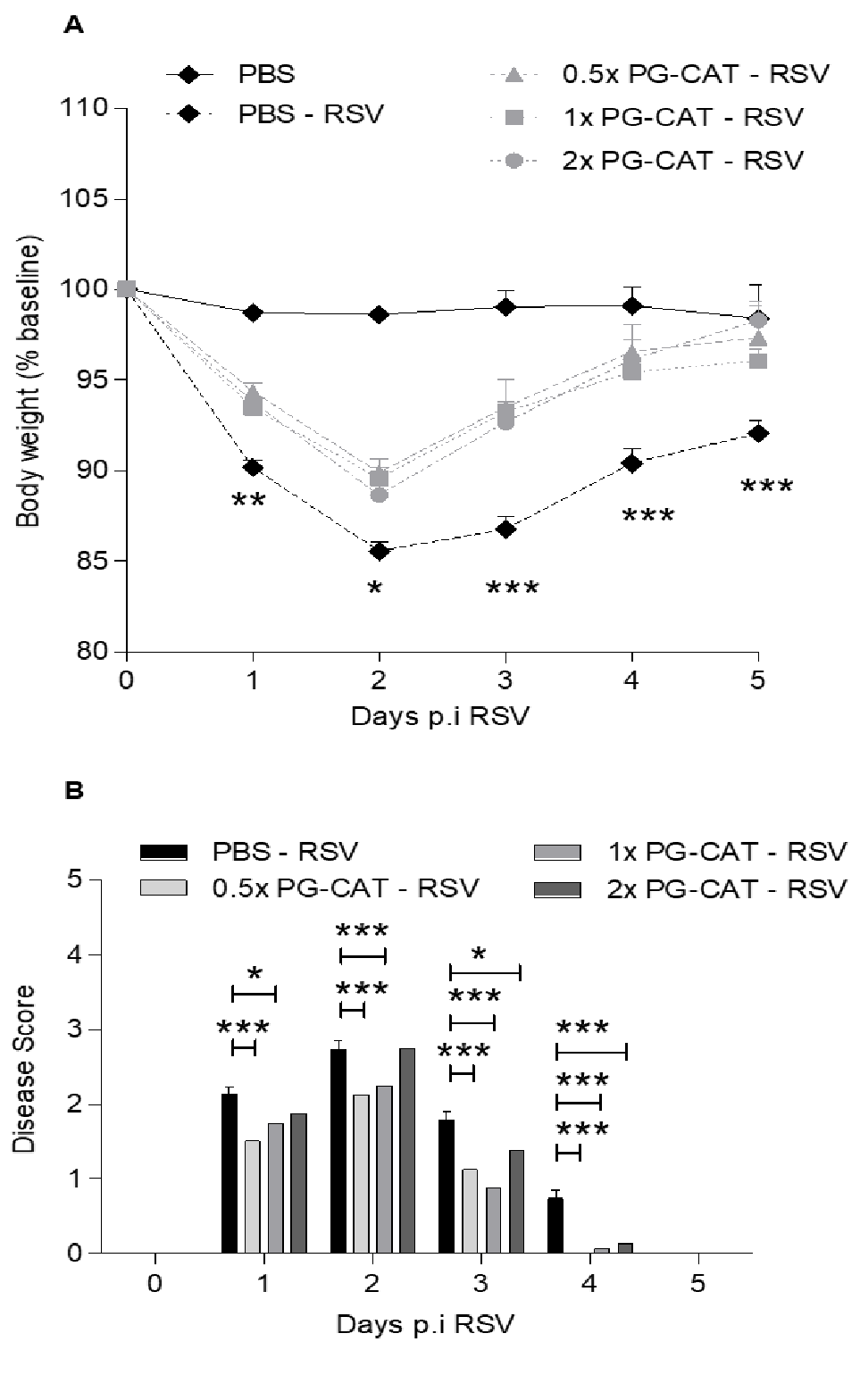

Supplement: Supplementary file 5 — Supplementary Figure 2. [file 41598_2020_60443_MOESM5_ESM.tif]

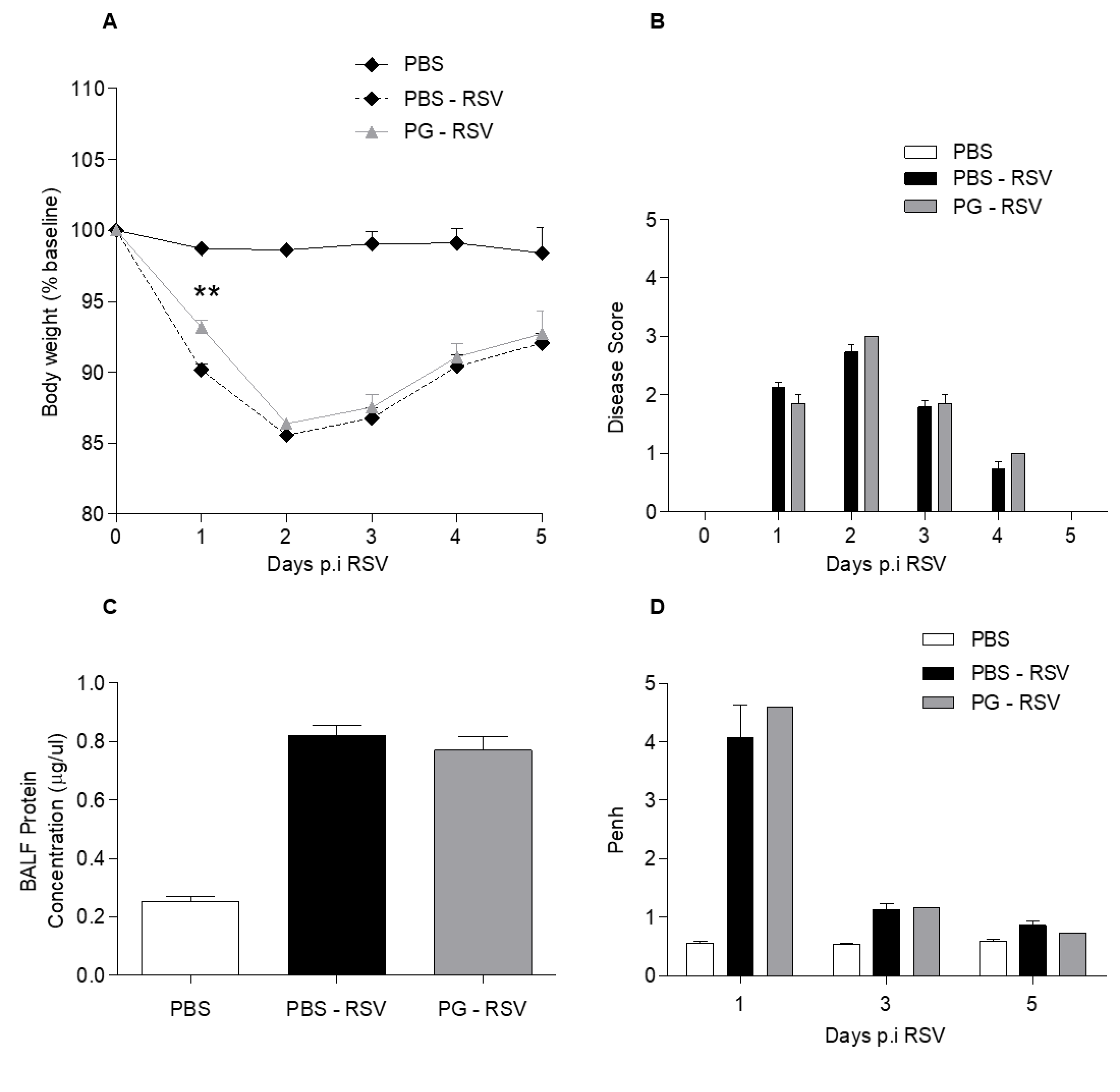

Supplement: Supplementary file 6 — Supplementary Figure 3. [file 41598_2020_60443_MOESM6_ESM.tif]

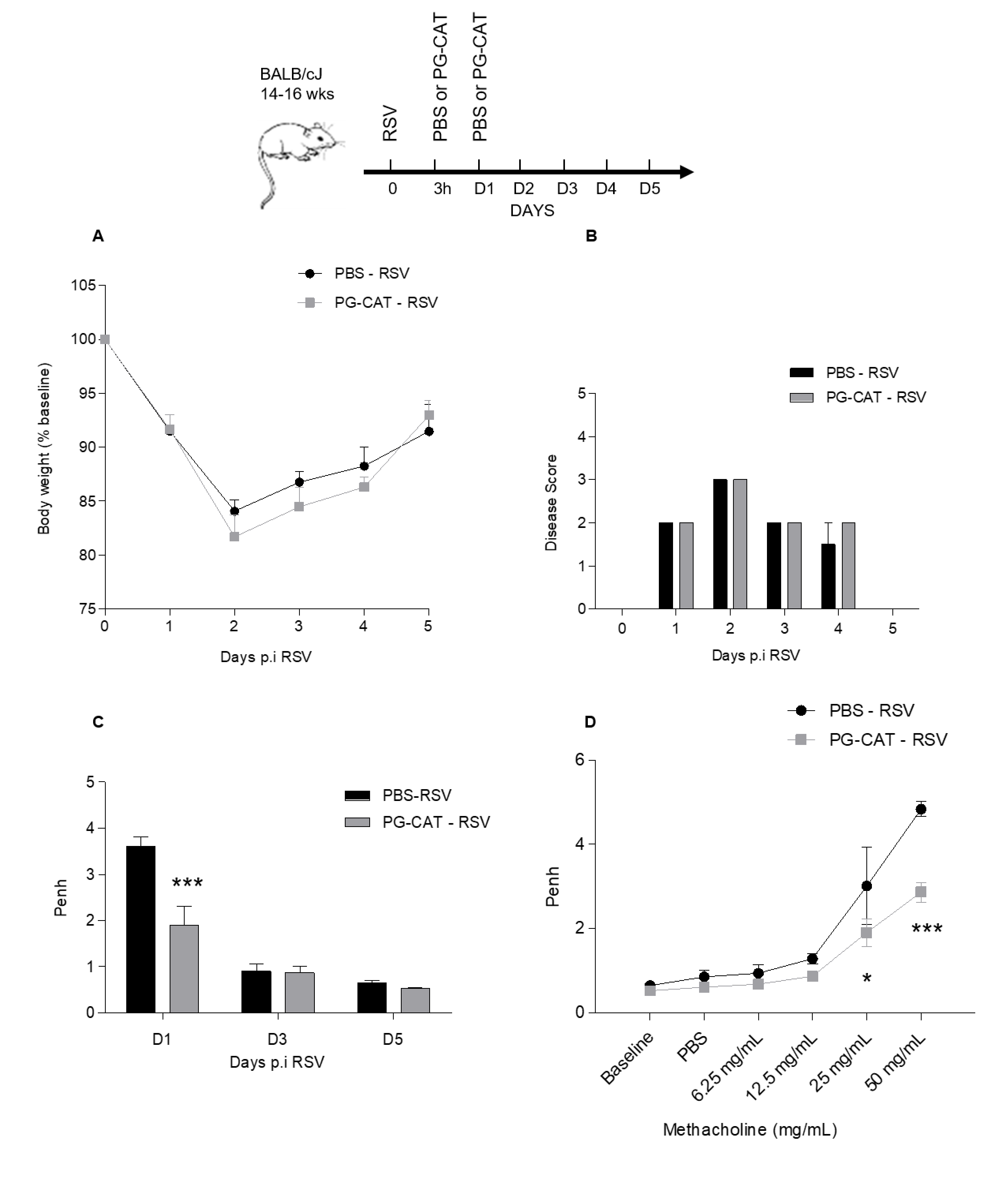

Supplement: Supplementary file 7 — Supplementary Figure 4. [file 41598_2020_60443_MOESM7_ESM.tif]
